# European Correspondence

**Published:** 1859-10

**Authors:** 


### EUROPEAN CORRESPONDENCE.

### ARTICLE VIII.

Paris, *June*, 1859.

Messrs. Editors:

Since writing my former letter, a successful effort has been made in England to form a faculty for a dental school, originating with the College of Dentists, which very naturally instigated the Odontological Society to follow a very good example. I have heard the names of the gentlemen composing these faculties, but have no remembrance of having seen them before as connected with any step, or as identified with the leading theories or science of the profession ; it is likely therefore that a mere local interest is desired.

As much as we can congratulate our English brethren for the unquestionable advance upon past conditions in this movement, we can only do so with two distinct objections to their present scheme for dental education. First, from an entire want of legal form or parliamentary power, they must be regarded as mere local, individual bodies, who have constituted themselves competent teachers of their professional brethren, who stand upon the same ground, with the same rights and privileges as they themselves, which will ever produce an antagonism in their business interest, anything but productive of additional success to the body of practitioners, or promotive of their position in the estimation of the world.

We cannot but think that had the proper application been made, by the proper persons to Parliament for this power, that it would not have been refused, did the profession hold the position in public estimation of a necessary and useful body. Looking upon this matter as a stranger, disconnected from any interested motive, it appears as indicative of a want of confidence either in the parties selected to fill their posts, or in the position in which the pursuit is estimated by the community at large, for did the calling hold a responsible and esteemed position, Parliament would have granted a reasonable and necessary demand, failing in this, the utter futility of an application would be but too evident, so that one is almost forced into the belief that a great fundamental want is too seriously felt in the profession as yet to have its efforts clothed with the sovereignty of law.

Second, being informed of the universal use of amalgams and cements as substances for filling teeth, and also of the general use of bone for mechanical pieces, we are induced to estimate the value of the education thus based, as of a very low grade, for although this is only a small portion of the curriculum of the dentist, yet it is the grand object of his pursuit and their perfection, the purposes of his study. I trust my English friends will bear with my frankness, in stating the singularity of their advocating the use of materials which our whole hemisphere repudiates. Such as is pronounced unnecessary by thousands of skillful practitioners, and which is admitted to be objectionable by its *best* advocates, should certainly induce more care and a closer scrupulousness in selection of material.

I am the more induced to make these remarks when I think how recklessly many of our practitioners perform various operations for the sake of the fee merely, feeling assured, that an agent at all admissible, so easily and rapidly worked, would induce its immediate adoption by them, did its usefulness or necessity justify them at all. It comes to a simple proposition: If the great majority of the best practitioners pronounce it objectionable and unnecessary, why should the minority continue its use? To one unengaged in practice, it would appear a sorry base to found an educational system upon; for one is so inclined to reason, that with these fundamental errors others of a greater or lesser magnitude must exist.

A great peculiarity of English practice is the immense amount of mechanical work done, you enter the laboratory and find hundreds of old plaster casts numbered and placed carefully away, for you must know that they, in making bone pieces, expect to furnish every two or three years a new set to the person, and it is no uncommon thing for orders to come from a long distance for sets of teeth, to be sent by express, as a tailor would a garment. Some employ as many as twenty journeymen in their work shops, who earn from £1 to £4 per week, and of course who make immense profit to their employers. From this you will readily perceive that mechanical work constitutes by far the largest amount of practice in that country, and may be the reason why they are so much in the background in filling, &c., for in the United States, it perhaps bears but an inferior proportion of the practice, certainly not the most profitable part. I have heard much complaint about their depots in this country, and doubt not there is much room for it. As every thing is dear and hard to get, London being the great centre, indeed I believe the only place of supply, this being done entirely by travelers. There is an effort at such an establishment in Glasgow, where I was told teeth could be bought cheaper than in the United States, after paying duties and charges, with whatever of commission or profit they may allow. How this is brought about I presume many users and vendors would like to know.

It so happened that while calling upon some of the

American practitioners in London, I was introduced to several of the leading noblemen of the kingdom, who, I was not uninterested to find, were wearing pieces of plate work made after the American system, and about which they were full of congratulation and satisfaction. Indeed, the simple and unpretending affability of the gentry every where in this kingdom, is something worthy of the greatest admiration, for although they pay a fee which to an American practitioner must be enormous, they seem, when their wants are well supplied, as overloaded with unrequited indebtedness.

I reverted in my former letter to the general use of chloroform in all surgical operations, and in midwifery, but I did not know of its very general use in the dental practice, which I have been informed is the case. My attention has been called to this fact from reading the account of another death from its use in the hospital for diseases of the eye. This case was one of strabismus, and of course was an operation of but trifling magnitude, and one which could have been accomplished safely and certainly without an anæsthetic, but its universality in this country was the cause of the unpardonable death of this poor victim.

The American dentist is much to be praised for his entire rejection of this agent, as one too fraught with danger to justify its use in his simple class of surgical cases, but more especially for the credit due to them for their adhesion to *ether*, as a preferable anæsthetic. Professor Simpson has certainly carried off all the praise for the introduction of chloroform, but I believe it is now certainly known that his attention was first directed to its use by one of the leading druggists of Liverpool. But thus accident robs the originator on this side of the water at times as upon ours.

I have paid some little attention to the instruments used in the country, especially the forceps, which, as compared with those of the United States, are small, weak, and illy adapted to the tooth for which they are designed, if teeth grow here as there, nor can they possess sufficient strength as required with us. They are, as every thing mechanical is here, very beautifully finished ; this remark may apply to all the dental instruments, which have a wondrous similar appearance to those I have been tortured with by my own dentist, and I suppose have been imported from time to time. This, however, is not generally recognized as a fact, but only so with a few individuals, who they are, or what use they make of them, some future time must enable me to state; at present my observation is altogether too limited, and common report is about as accurate here as elsewhere. I presume, therefore, you are as well informed as I am at this time, about manipulative abilities, &c.

Nothing can be more pleasant than the trip from London to Paris, provided always, you are not sea sick in crossing the channel, that you understand French well, and that your passport has been *viséd* by the French and American consul.

The rail road coach in England is built, that is the first class ones, for comfort and convenience, if you can forget the fact of being locked in until you arrive at a station, when you can regain your liberty, to be locked in again and again, until you reach your destination.

You would be astonished to find people who have crossed and re-crossed the broad Atlantic without an approach to illness, suffer this worst of all misadventure, sea sickness, in passing this little body of troubled waters. Indeed, it was told me, that this accounted for Napoleon the first not having conquered this garden island, as he was a bad sailor, and always sick on the channel, and ever cared particularly for his soldiers, and from much observation I am sure the same trouble will deter the present great man from attempting it, for he would certainly be sick of his trip before crossing this detestable little sea.

The people think those *matella towers* would have a good deal to do with it, but the world will never be gratified with opportunity of determining.

Bologne Sur Mer is a pretty little town with a beautiful harbor, celebrated by Napoleon's various fortifications, and by a beautiful shaft erected to his memory. I ascertained that it enjoyed the luxury of several dentists, giving them a fair and lucrative practice. Has a fine hospital, with one of the largest and most beautiful cathedrals on the continent, highly ornamented by intensely chiseled masonry, and decorated with vast and expensive paintings. Although an ordinary day, it was filled by the devotees to this prevailing religion, who seemed principally to belong to the working classes. Having secured a night's lodging, and a good breakfast, I started *en route* for Paris, but to my shame and astonishment, my trunk was carried to the depot on the back of a woman, whilst the kind porter walked by her side. This, I ascertained, was the custom of the country, men thinking it unmanly to do such work whilst women are to be had. It is not uncommon to see women and also men, absolutely in a species of harness, attached to a go-cart, drawing great loads through the city.

Designing to remain a season in France, and to go from thence to Belgium, I will have the pleasure of writing you from the latter place, giving whatever I can glean, as interesting to the dental profession.

